# Effectiveness of flipped teaching on the knowledge and self-efficacy of nursing personnel in non-pharmacological pain management-aromatherapy: a quasi-experiment

**DOI:** 10.1186/s12912-022-01042-6

**Published:** 2022-09-19

**Authors:** Ching-Wen Chiu, Chieh-Hsing Liu

**Affiliations:** 1 Department of Health Promotion and Health Education, National Taiwan Normal University Taipei, New Taipei City, Taiwan, R.O.C.; 2 Department of Nursing, Taipei Medical University Hospital, Taipei, Taiwan, R.O.C.; 3 Department of Health Promotion and Health Education, National Taiwan Normal University, 5F., No. 17, Tanxing St., Shulin Dist., 238002 New Taipei City, Taiwan, R.O.C.

**Keywords:** Aromatherapy, Flipped teaching, Knowledge, Self-efficacy, Pain, Pain management, Essential oils

## Abstract

**Background:**

Aromatherapy is effective in treating pain; however, aromatherapy is not offered in formal nursing education in Taiwan. This study designed aromatherapy training courses for nurses using the flipped teaching approach and explored the effectiveness of the method, which can serve as a reference for future aromatherapy courses.

**Methods:**

A quasi-experimental design and convenience sampling were adopted. The participants were nurses who had been employed for over one year in two hospitals in Taiwan. Forty nurses were included in the experimental and control groups. The intervention of this study was performed in February 2020. Nurses in the control group received one hour of introduction to the use of aromatherapy in pain management. This class was delivered using the traditional teaching method, during which a researcher served as the lecturer. Nurses in the experimental group received a 2.5-h flipped teaching course on aromatherapy. Two weeks before classroom activities, the nurses in the experimental group watched a 30-min aromatherapy concept video on an e-learning teaching platform. Thereafter, the nurses participated in two hours of classroom teaching in both groups. The course design included group discussions, mind mapping, case discussion, practice with essential oils, and do-it-yourself essential oil preparation.

**Results:**

Pre- and post-test knowledge and self-efficacy in aromatherapy were assessed. There were no significant differences in the pre-test knowledge and self-efficacy scores between the two groups. The test was analyzed using a generalized estimating equation. Post-test knowledge and self-efficacy results showed that the change in scores in the experimental group was significantly better than that in the control group, indicating that flipped teaching improved the participants’ knowledge and self-efficacy in aromatherapy.

**Conclusions:**

This study confirmed that flipped teaching was effective in helping nursing personnel learn aromatherapy. Implementation of aromatherapy by nurses in clinical practice and its impact on patient care should be further assessed.

## Background

Pain is one of the most common medical issues for patients. Lack of pain relief does not only lead to physical imbalance but also affects the daily life, sleep, mood, and quality of life of patients, which is detrimental to patient recovery [1]. Pain management is divided into two categories, drug treatment and non-drug treatment. Many studies have listed non-drug pain countermeasures as necessary and that they can reduce the dosage of painkillers and improve patients’ anxiety and depression to achieve patient-centered holistic pain care. As a non-pharmacological pain management method, aromatherapy features convenience and few side effects [2, 3]. Aromatherapy refers to the use of essential oils to prevent and treat diseases through the uptake of these oils via the olfactory organs or skin. Through massage, bathing, inhalation, spraying, and other methods, essential oils enter the human body to generate positive emotions and activate the inborn self-healing capacity of humans, thereby relieving mental stress and improving physical health [3]. Many studies have indicated that aromatherapy could be combined with traditional therapy in pain treatment. Aromatherapy is suitable for various types of pain, such as pain from obstetric, gynecological, musculoskeletal, and neurological diseases and from surgery [4–8]. In aromatherapy, appropriate essential oils are often chosen for different causes of pain and are combined with massage for relaxation; their pleasant smell can also help relieve chronic pain [9]. Aromatherapy has been shown to be applicable in medical care. To integrate the safe application of aromatherapy into nursing practice, its knowledge and application must be incorporated into nursing education. However, aromatherapy-related courses have not been universal in nursing schools in Taiwan, with only the introduction of aromatherapy included in the section of pain care assessment and management [10]. A previous study has indicated that nursing staff generally have insufficient knowledge on aromatherapy, and this significantly correlated with the attitude toward aromatherapy. On this basis, the authors have suggested that nursing staff be provided with education and training to provide them with relevant knowledge regarding the correct use of essential oils related to aromatherapy [11]. In addition, although previous pain management training for nursing staff used to be carried out with a unidirectional teaching approach, related literature has mentioned that this should be strengthened with case discussions or simulation practices to enhance learning effectiveness. Therefore, “flipped learning” has been introduced into the in-service education of nursing staff.

The concept of flipped teaching originated in the United States [12]. In flipped teaching, students read the teaching materials provided by the instructors before a class and write down key points or questions. The classroom is changed from the traditional one-way teaching to student-centered two-way learning activities such as discussions, debates, group discussions, and case reports. The instructors motivate the students, answer questions, clarify confusions, etc. in the classroom to help students establish correct concepts, stimulate thinking, guide them in problem-solving, and encourage students in knowledge application [13]. The four pillars of flipped learning are F (Flexible learning environments), L (Learning culture), I (Intentional content), and P (Professional educator) [14]. Multiple domestic and foreign studies have utilized flipped learning in nursing education and demonstrated that it can promote positive responses in cognition, skill, attitude, self-efficacy, and learning satisfaction [15, 16]. The flipped learning is an opportunity for increased contact time between faculty and students. Learners clarify and apply previous learning, connect this learning to clinical case scenarios, and apply classroom teachings to their work in the clinical setting. In addition, a “flipped classroom” can spark interest in higher education and offer a student-centered learning approach, which satisfies the existing needs of nursing staff and improves their learning motivation. This teaching method is consistent with the requirements for clinical nurses [12, 17, 18]. Many domestic and foreign studies have applied flipped teaching in nursing education, which includes all nursing fields [15].

Self-efficacy is a core concept proposed by the American psychologist Bandura as a part of the social cognitive theory in 1982. It refers to an individual’s belief in his or her capacity to execute behaviors necessary to produce specific performance attainments, and it is an important factor in maintaining or changing one’s health behaviors. Self-efficacy emphasizes that, while relevant knowledge and skills are necessary, an individual must also have adequate self-confidence and believe that they have the capacity to carry out relevant behaviors in order to do so [19]. Self-efficacy can be affected by four factors, namely, past performance, vicarious experiences, verbal persuasion, and physiological cues [20]. Various studies on learning and self-efficacy have verified that the latter can affect an individual’s learning motivation and behavior [21]. Therefore, improving nurses’ self-efficacy can make them believe that they are motivated and skilled; thus, they will act on achieving their goals. On the contrary, individuals with low self-efficacy tend to expect negative results, which leads to declining learning motivation, passive learning, and the inability to use resources to take necessary action. Self-efficacy is the decisive factor in determining one’s behavior motivation and can be escalated by past performance, vicarious experiences, verbal persuasion, or physiological cues. Consequently, the integration of these four elements into education and training courses will inspire nursing staff to perform specific nursing behaviors and improve their self-efficacy therein.

There have been many studies on the application of aromatherapy in clinical care, especially in pain management [3]. However, few studies have investigated aromatherapy education for nursing personnel in Taiwan [22]. Only one study investigated the learning outcomes of 2–3-year college students in applying essential oil preparations in aromatherapy for primary dysmenorrhea through problem-based learning curriculum design [23]. Therefore, the purpose of this study was to apply the flipped teaching approach to design teaching and training courses in aromatherapy for nursing personnel and to explore the effectiveness of the method among nursing personnel, which may serve as a reference for nursing schools or medical institutions to plan future teaching and training courses in aromatherapy.

## Methods

### Study design and study participants

**Fig. 1:**
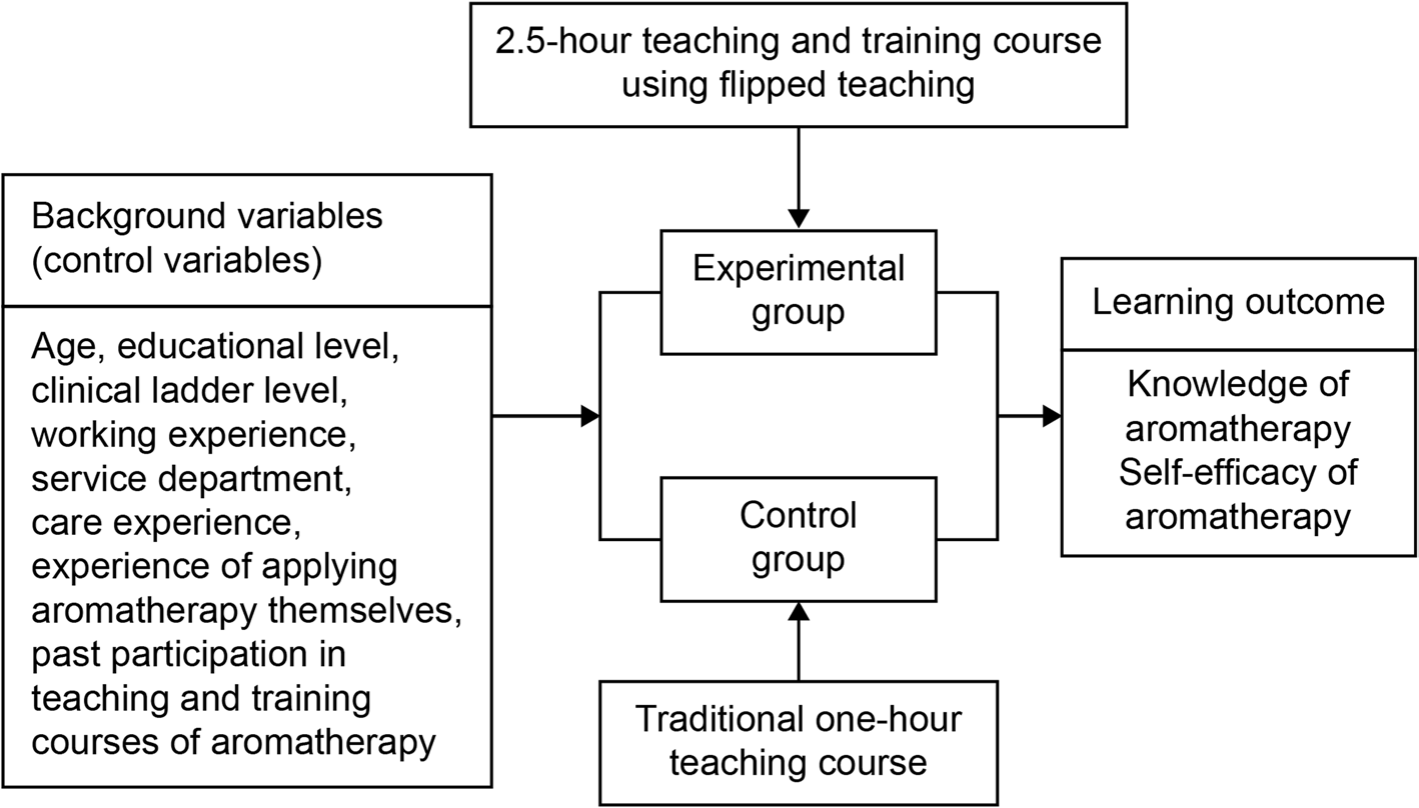
Study design

In this study, a quasi-experimental design was applied with pre- and post-test assessments in the experimental and control groups. Convenient sampling was adopted to publicly recruit nurses who were willing to participate in the study from two hospitals of the same medical system in northern Taiwan. The inclusion criteria were nurses who had more than one year of experience in medical or surgical wards. Conversely, those who did not engage in the full study, failed to complete the questionnaire, or provided invalid responses were excluded. The sample size was estimated using the G Power 3.1 software, with error (α) = 0.05, test power = 0.8, and effect size = 0.8. The estimated sample size was at least 26 participants in each group. A total of 80 participants, 40 in the experimental group and 40 in the control group, were included. The experimental group consisted of 40 nurses, selected from a total of 500 nurses from Hospital A, who received 2.5 h of flipped education on aromatherapy. The control group consisted of 40 nurses, out of 800 nurses from Hospital B, who underwent one hour of traditional aromatherapy education and training (Fig. 1).

The pre-test was completed before the two groups began the course, and it included basic information and a questionnaire on the knowledge and self-efficacy of nursing personnel in aromatherapy. The post-test was conducted after the two groups had completed their respective courses, and it included a survey on the knowledge of and self-efficacy in aromatherapy. This study was reviewed and approved by the institutional review boards of the recruiting hospitals (No. CGH-P108116) before recruiting the participants. All participants signed a written informed consent form before participating in the study. Prior to data collection, researchers first explained the process, purpose, plan, and method of the study as well as the participants’ rights and interests. In addition, during the research process, the participants’ reactions were closely monitored to ensure that they were comfortable and without stress. Nurses had the right to refuse to participate or withdraw at any point of the study without compromising their work rights.

### Study instruments

#### Questionnaire on basic information

The questionnaire on basic information included basic demographic characteristics (age, educational level, work hospital, work experience, and clinical ranking), frequency of caring for patients with pain issues in the past, participation in aromatherapy courses, and experience in applying aromatherapy.

#### Knowledge and self-efficacy scale of aromatherapy for nurses

The knowledge section of the questionnaire included pain relief mechanism of action, route, dosage, and other aspects of aromatherapy for pain relief. It consisted of six true-or-false questions. Participants would score 1 for each correct answer and 0 for each incorrect answer. The self-efficacy section of the questionnaire included two items: “The level of confidence I have in providing aromatherapy information to patients when they are in pain,” and “The level of confidence I have in helping patients performing aromatherapy when a patient is in pain.” Each question is scored on a 5-point scale, with 5 indicating “extremely confident”, and 1 indicating “extremely unconfident”. The higher the score, the more confident the subject is in executing each behavior. Once the first draft was prepared, seven experts and scholars in related fields, such as health promotion and education, healthcare, and education, were invited to conduct content validity verification, utilizing the content validity index (CVI), Cronbach’s α, difficulty of questions, and degree of discrimination. They suggested that the CVI of the scale was 0.93. Internal reliability analysis during preliminary testing showed that Cronbach’s α was 0.91. The individual difficulty of each question ranged between 0.5 and 0.75 with an average of 0.64, indicating a moderate overall difficulty. Similarly, the individual discrimination degree of each question ranged between 0.45 and 0.8 with an average of 0.65, indicating an appropriate overall discrimination degree.

#### Introduction to the flipped teaching aromatherapy course

The flipped aromatherapy course was based on the four pillars of flipped learning (F, L, I, and P) [24], and incorporated the four elements of self-efficacy (past performance, vicarious experiences, verbal persuasion, and physiological cues) in the design. The class was delivered in two stages, namely pre-class self-learning and during-class teaching.

Each element of flipped learning was applied as follows:

1. Flexible environments: Students were asked to perform self-learning before the class commencement using electronic products, which was not limited by the environment, equipment, or time. Subsequent classroom teaching was carried out in groups, during which the traditional teacher-centered approach was replaced with student-centered group discussions.
2. Learning culture: The nursing staff were asked to watch a 30-min aromatherapy concept video on the e-learning teaching platform before participating in the flipped teaching course and acquaint themselves with the prerequisite knowledge of aromatherapy. Group discussions were designed in the classroom, allowing participants to achieve self-efficacy theory achievement performance and substitute experience and verbal persuasion through group discussions. The physical operation of aromatherapy essential oils was designed, partners were invited to provide feedback at the same time, and emotional stimulation of self-efficacy theory was achieved through personal experience.
3. Intentional content: The course was delivered using two teaching methods. The first one involved the design of group discussion activities based on the self-efficacy theory. Through concept clarification, case discussion, and mindmapping, this method equipped participants with the ability to prepare essential oils and strengthened their self-efficacy in performing aromatherapy on patients experiencing pain. The second approach involved practical and personal experience, providing participants with multiple essential oils to become familiar with their smells.
4. Professional educator: The researcher was the teacher of the pre-class self-study course, the leader of the classroom activities, and responsible for conducting group discussion and skill guidance, and the team members discussed the case together in the course. The researcher had 10 years of clinical teaching experience, received 48 h of training for the intermediate certification of aromatherapists, and practical experience in using aromatherapy in clinical practice.

#### Course activities

**Fig. 2:**
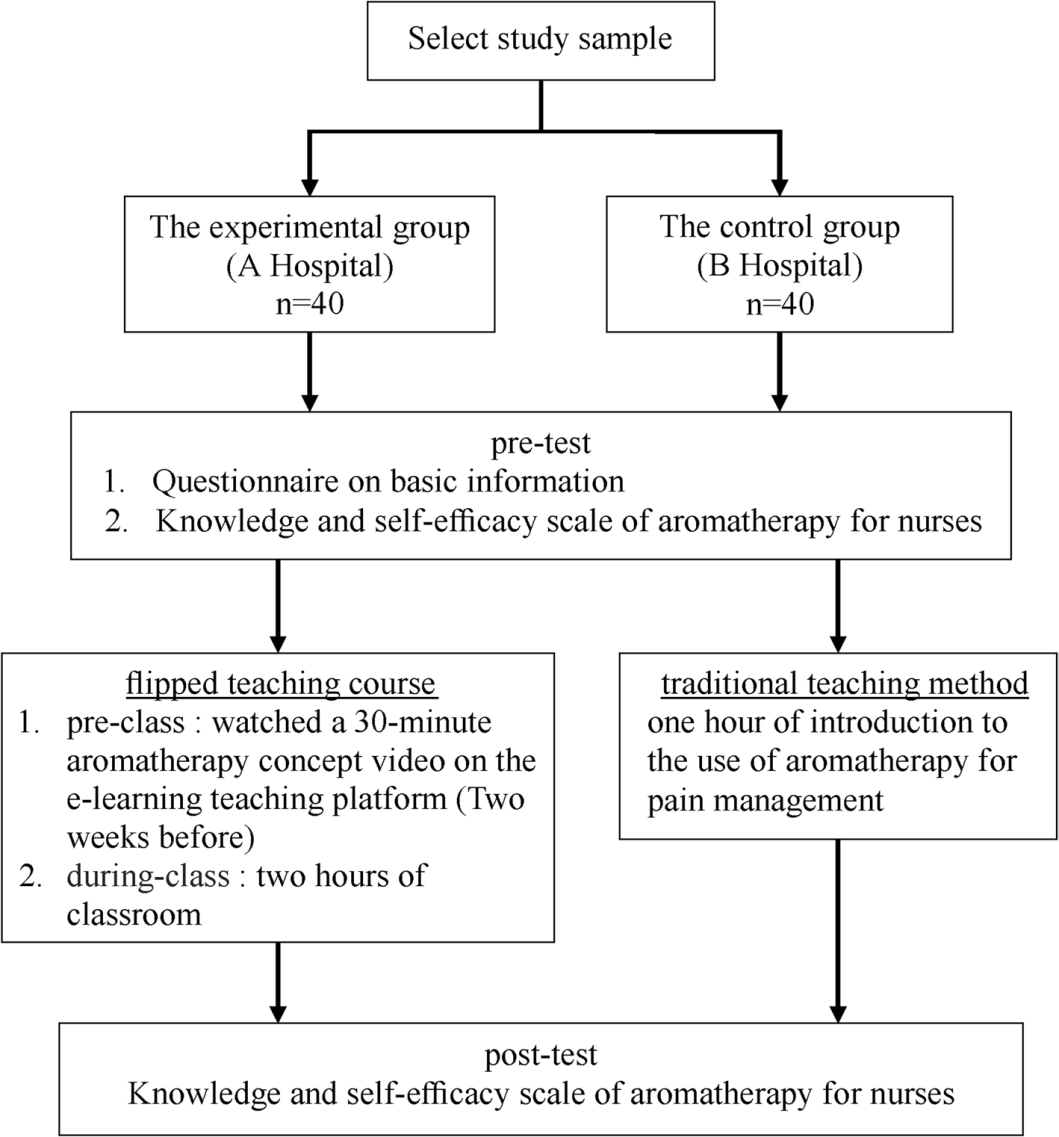
Flow chart of flipped education course intervention

The intervention of this study was performed in February 2020. Participants in the control group received one hour of introduction to the use of aromatherapy for pain management. This class was delivered using the traditional teaching method, during which a researcher served as the lecturer and delivered the content to the 40 nurses in the group. Contents of the class included the origin of aromatherapy, evidence of the role of aromatherapy in pain relief, types of essential oils to be used for different body systems, the mechanism of action, way of use, preparation of dosage, and precautions in using essential oils. Nurses in the experimental group received a 2.5-h flipped teaching course on aromatherapy. Two weeks before the classroom activities, the nurses in the experimental group watched a 30-min aromatherapy concept video on the e-learning teaching platform. The video included the following information: (1) the origin of aromatherapy; (2) evidence of aromatherapy and pain; (3) types of essential oils that can be selected for various systems of the body; (4) the best approach for application (topical, diffusion, or concentrated inhalation); and (5) precautions while using essential oils. Then, the nurses participated in two hours of classroom teaching in groups. The course design included a brief 15-min introduction of the self-study contents by the lecturer as a pre-class review for the students, 15 min of group discussions to clarify the concepts and share experiences, 30 min of mind mapping and case discussion, 20 min of practice with essential oils, and 40 min of do-it-yourself (DIY) essential oil preparation (Fig. 2).

### Statistical analysis

The Statistical Program for SPSS version 23.0 (SPSS Inc. Chicago. IU, USA) for Windows was used for the statistical analysis, and a statistical significance level (α) of 0.05 (two-tailed test) was applied. Descriptive statistics of the background variables of the study participants were calculated, and the results are presented as frequency distribution, percentage, mean, and standard deviation. Subsequently, an independent sample t-test, chi-squared test, and generalized estimating equations (GEE) test were performed to understand the difference in the knowledge of and self-efficacy in aromatherapy between the experimental and control groups.

## Results

### Analysis of background variables of the study participants

A total of 80 participants were included in this study, with 40 each in the experimental and control groups. All the participants were female, with a mean age of 28.87 ± 5.64 years and an average work experience of 6.26 ± 5.45 years. Most participants had received university education (56 nurses, 70%). In terms of working institutions and departments, 38 participants (47.5%) worked in the internal medicine department, 24 (30.0%) in the surgical department, and 18 (22.5%) in intensive care units. In terms of the clinical nursing ranking, most nurses were N2 (34 nurses, 42.5%). Most nurses (39, 48.75%) provided care to patients with pain daily. A total of 23 participants (28.75%) had attended aromatherapy courses in the past year, including 10 (25.0%) in the experimental group and 13 (32.5%) in the control group. Nine participants (11.25%) had used aromatherapy on themselves when they had experienced pain in the past, including five (12.5%) in the experimental group and four (10%) in the control group. The difference between the background variables of the two groups were assessed by the independent sample t-test and the chi-square test. No significant differences were observed in age, work experience, education level, working institutions, clinical ranking, and the frequency of taking care of patients with pain at work within the past six months (*p* > 0.05), indicating the homogeneity between the two groups of participants.

### Comparison of the knowledge and self-efficacy of the study participants in aromatherapy

For the pre-test knowledge scores of the two groups, the mean score of the experimental group was 4.65 ± 1.05 and that of the control group was 4.05 ± 0.99. There was no significant difference in the pre-test knowledge scores between the two groups. For the post-test knowledge scores, the mean score of the experimental group was 5.53 ± 0.56 and that of the control group was 4.38 ± 1.15. The post-test knowledge score of the experimental group was 1.15 points higher than that of the control group, with significant differences between the two groups (*p* < 0.05) (Table 1).

**Table 1:** Differences between pre-test and post-test knowledge and self-efficacy scores of participants in aromatherapy (*N* = 80)

**Variable**	**Pre-test**			**Post-test**		
	**Mean ± SD**	**T**	***P***	**Mean ± SD**	**T**	***P***
Knowledge						
Experimental group	4.65 ± 1.05	1.117	0.267	5.53 ± 0.56	8.249	0.00*
Control group	4.05 ± 0.99			4.38 ± 1.15		
Self-efficacy						
Experimental group	7.03 ± 1.12	0.787	0.434	8.55 ± 0.96	6.12	0.00*
Control group	6.83 ± 1.52			6.90 ± 1.41		

^*^p < 0.05

Regarding the participants’ self-perceived capacity (self-efficacy) in performing aromatherapy for pain management, the pre-test self-efficacy of the experimental group was 7.03 ± 1.12 and that of the control group was 6.83 ± 1.52. The self-efficacy of the two groups was only at the normal level, with no significant inter-group differences. The post-test self-efficacy score was 8.55 ± 0.96 in the experimental group and 6.90 ± 1.41 in the control group. A significant difference in the post-test self-efficacy scores was observed between the two groups (*p* < 0.05) (Table 1).

### Analysis of the effect of teaching and training interventions using flipped teaching

The research hypothesis that “nurses in the experimental group receiving flipped teaching intervention are significantly better than those in the control group in terms of knowledge and self-efficacy in performing aromatherapy” was tested; GEE was used for the analysis. The results regarding knowledge showed an interaction term between “group” and “pre-test/post-test,” indicating that the change in knowledge scores of the experimental group was significantly better than those of the control group (B = 1.200, *p* < 0.001). This showed that flipped teaching and training improved the nursing personnel’s knowledge of aromatherapy. The self-efficacy results showed that there was an interaction term between “group” and “pre-test/post-test,” indicating that the change in self-efficacy scores of the experimental group was significantly better than those of the control group (B = 1.450, *p* < 0.001). This showed that flipped teaching and training improved the self-efficacy of nursing personnel in performing aromatherapy (Table 2).

**Table 2:** Effect analysis of the intervention of teaching and training using flipped teaching (*N* = 80)

Variable	*B*	Standard deviation	95% CI	Wald chi-square	*P*
Knowledge					
Intercept	4.375	0.179	4.024–4.726	596.107^**^	< 0.001
Group	0.275	0.243	-0.201–0.751	1.281	0.258
Pre/post-test	-0.325	0.119	-0.559–-0.091	7.420*	0.006^*^
Group × pre/post-test	1.200	0.201	0.787–1.613	32.382**	< 0.001
Self-efficacy					
Intercept	6.825	0.185	6.462–7.188	1356.654	< .001
Group	0.200	0.262	-0.314–0.714	0.582	0.445
Pre/post-test	0.075	0.189	-0.297–0.447	0.157	0.692
Group × pre/post-test	1.450	0.268	0.925–1.975	29.250	< 0.001

^*^ *p* < 0.05

^**^ *p* < 0.001

## Discussion

This study found that only 28.8% of the participating nursing staff had received education and training on aromatherapy, and only 11.3% had previous experience in personally using aromatherapy to manage their pain. This result is similar to the findings of Laio et al*.* [10], who mentioned that only 32.3–36.6% of nurses received related education and training from school or workplace, and only 28.1% used aromatherapy themselves. Therefore, insufficient education on aromatherapy is currently provided by nursing schools or clinical nursing education courses. Aromatherapy was introduced in Taiwan in the late 1980s. Although many studies have confirmed the effectiveness of aromatherapy in treating pain and that it is one of the adjuvant therapies available to nursing personnel clinically, aromatherapy has not yet been included in formal nursing education, and nursing personnel are not familiar with aromatherapy. Therefore, aromatherapy teaching and training should be planned and promoted in the future so that nurses can assist patients in receiving appropriate aromatherapy during clinical care [3, 22, 25].

This study integrated a systematic literature review to design the teaching plan for the flipped teaching course [26]. Along with students’ self-learning of the prerequisite knowledge of aromatherapy before class, group discussions were also included in the course design to clarify concepts and share personal experiences of aromatherapy in life or work, which may deepen the students’ understanding of aromatherapy and promote their motivation to use it. During the class, the lecturer prepared over ten kinds of essential oils commonly used for different types of pain so that students could smell the essential oils themselves, which deepened their understanding and memory of different essential oils. Case discussion using mind mapping may help to summarize the systemic problems of the case in combination with the use of essential oils. Additionally, the group discussion may help the students brainstorm to acquire the ability to treat patients using aromatherapy. As previous investigators have reported, group learning in nursing education is a commonly used teaching method in a flipped classroom, and group learning motivates students to learn. Classroom discussions were aimed to solve clinical problems students may face in the future and develop their professional skills, thereby improving their clinical care capacity and critical thinking skills [27]. Finally, each student was asked to develop DIY essential oils according to various pain conditions, to enhance their self-efficacy in aromatherapy.

The results of this study indicated that the post-test scores of the experimental group were not only higher than pre-test scores, but also higher than the post-test scores of the control group, the differences of which were statistically significant. The fact that the post-test self-efficacy of the experimental group was substantially higher than that of the control group suggests that the perceived capacity of the experimental group for performing aromatherapy was considerably escalated after flipped learning. By contrast, after they received traditional education, although the knowledge and self-efficacy scores of nurses in the control group increased, only insignificant differences were observed. It can thus be concluded that, through flipped learning for aromatherapy, nurses can master non-pharmacological pain management in a relatively short timeframe. This reveals that the flipped learning model is superior to traditional teaching approaches and is effective in improving students’ knowledge and self-efficacy [28–31].

### Limitations

Due to the limitations of time, manpower, and material resources, as well as to prevent mutual contact or interference between the experimental group and the control group in the same hospital, this study selected participants for the control group from another hospital in the same medical system. However, participants in both the experimental and control groups received the same education and training prior to the study. The control group received educational training for 1 h. Because of the design of the course activities, the experimental group received a 2.5-h flipped education course. The inconsistent timing of educational training between the control and experimental groups is also a limitation of this study. There was no difference in previous participation in aromatherapy courses between the two groups of participants, and thus, no difference in knowledge of aromatherapy in the pre-test between the two groups. Furthermore, limited by the small sample size, the investigators set the criteria to mainly include nursing personnel for adult patients, making it impossible to generalize the findings to all nursing personnel. Despite these limitations, this study was still the first in Taiwan to explore the effectiveness of flipped teaching on the nursing personnel’s knowledge of and self-efficacy in aromatherapy and may serve as a reference for future course designs.

## Conclusions

Aromatherapy courses have not been universally taught in nursing departments and medical institutions in colleges and universities in Taiwan, and nursing personnel have not been fully educated in aromatherapy. Nurses may only attend aromatherapy workshops based on a personal decision and at their own expense. These factors have greatly limited nurses’ ability to apply aromatherapy to pain management. It is recommended that aromatherapy be included in educational training courses in pain management. The results of this study confirmed that the use of the flipped teaching approach indeed improved the knowledge and self-efficacy of nurses. Therefore, we hope that the course design in this study may serve as a reference for the future design of aromatherapy courses in nursing schools or medical institutions. This study only discussed the learning outcomes after the educational intervention. We recommend future follow-up on the implementation of aromatherapy by nursing staff in clinical practice and on the effect of pain relief on patients in order to improve the quality of patient care.

## Data Availability

The datasets used and/or analyzed during the current study are available from the corresponding author on reasonable request.

## Abbreviations

CVI: Content validity index
DIY: Do-it-yourself
GEE: Generalized estimating equations
